# Narrative review: Continuous professional development training programmes in Africa and their limitations

**DOI:** 10.4102/ajlm.v14i1.2602

**Published:** 2025-05-16

**Authors:** Nqobile Ndlovu, Rajiv T. Erasmus, Annalise E. Zemlin

**Affiliations:** 1 Division of Chemical Pathology, Faculty of Medicine and Health Sciences, Stellenbosch University, Cape Town, South Africa; 2 African Society for Laboratory Medicine, Johannesburg, South Africa; 3 South African Medical Research Council (SAMRC) Research Unit on Cardiometabolic Health, Cape Peninsula University of Technology, Cape Town, South Africa; 4 National Health Laboratory Service, Tygerberg Hospital, Cape Town, South Africa

**Keywords:** Continuing professional development, laboratory professionals, training, African countries, health professional boards

## Abstract

Continuous professional development (CPD) represents a cornerstone in the advancement of professional skills and knowledge across various sectors. It is globally recognised as a transformative process that unlocks potential, increases capacity, and fosters personal growth. This narrative review article aimed to understand how the CPD training programmes for laboratory professionals are implemented and sustained in Africa. A narrative review was conducted where a comprehensive search was conducted across PubMed, Embase, and web searches for white and/or grey literature, facilitated by a custom Python script. A combination of keywords, truncations, and subject headings targeted four key themes: Continuing professional development (and related terms), laboratory professionals, African countries, and aspects of implementation and scoring. The search was restricted to articles in English published from 2009 to 2024. While the actual training needs and gaps for CPD programmes are widely known, the actual implementation of CPD has remained a challenge. In the past, CPD training programmes have been implemented to address the lack of skills and the insufficient and skewed distribution of these health workers. This approach is not sustainable and has led to some challenges with coordination, quality assurance, and regulation. Each country has its unique context and training needs; therefore, CPD needs to be more coordinated and tailored so that professionals are given the right training for their needs.

**What this study adds:**

Addressing training gaps for laboratory professionals in Africa will require a well-structured, coordinated and tailored approach that will deliver a continent-wide CPD programme.

## Introduction

Medical laboratory professionals play a key role in the detection, diagnosis, and monitoring of diseases in today’s healthcare environment.^1,2^ They are the backbone of quality diagnostics, providing essential services that range from routine testing to specialised analyses critical for patient care.^3,4^ Their work informs clinical decisions, guides treatment, and contributes to disease surveillance and control by allowing correct diagnosis, proper management of patients, and also by improving public health outcomes.^1,5^ Despite this, the African continent continues to grapple with an inadequately skilled and competent laboratory workforce.^3,6,7,8,9^

With the growing need for a competent work force, health professional boards around the globe are increasingly demanding that practitioners demonstrate their participation and commitment to continuous professional development (CPD). It will, therefore, be important that opportunities for CPD be made available and relevant to the cadre of healthcare professionals.^10,11,12^ Basic education and training alone are no longer enough, as knowledge is rapidly evolving and changing.^13^

It is common practice, in many countries, that laboratory professionals need to be registered and licensed to practise – although implementation has varied across the African continent. Registration and licensure should be structured in a manner that includes new knowledge acquisition, skills and competence improvement given the ever-changing landscape of medical practice and technology. To promote and encourage the culture of learning, developed countries have adopted new strategies where remuneration increases and career growth for medical laboratory professionals are linked to participation in CPD programmes.^14^

### Understanding the role of continuous professional development programmes

Continuous professional development represents a cornerstone in the advancement of professional skills and knowledge across various sectors, and is globally recognised as a transformative process that unlocks potential, increases capacity and fosters personal growth.^15,16,17^ It provides an important opportunity for progressing and developing one’s career. Health professionals will need to continuously advance their knowledge, learn new competencies, and build on existing ones.^18,19,20^ The main purpose of CPD is to promote better patient care and satisfaction by ensuring that health professionals are knowledgeable and empowered with latest skills.^21,22,23^ In some African countries, there is a lack of opportunities and structures that will ensure that learning for medical laboratory professionals is happening routinely and continuously.^10,24,25,26^ The implementation of CPD in Africa varies, with some regions developing robust policies and frameworks, while others are still in the nascent stages of policy development.^27,28,29^ In some places where these programmes exist, they are fragmented, neither curated nor accredited, and eventually no recognition is provided to learners to contribute towards their CPD.^30^ For many laboratory practitioners, the only training events available for CPD are the *ad hoc* peer-to-peer trainings organised by employers.^10^ To compound the situation, there is little to no monitoring or evaluation of CPD implementation to understand what works and what does not. As such, both policymakers and those responsible for resource allocation are left with limited evidence to support scale-up of CPD programmes. In Europe, for example, the CPD programme is well structured, and this has been demonstrated by a CPD programme established among the 13 members of European Federation of Clinical Chemistry and Laboratory Medicine, with CPD credits being used for renewing the practising licence.^31^

One of the success factors for the implementation of CPD is that it should be driven by laboratory professionals themselves, employers, and training institutions, and should be well organised by national professional institutions, who are able to deliver well-structured CPD activities.^24,31^ Countries have different levels of institutional support for promoting and encouraging human resource development through CPD programmes, and studies have shown that there is a low participation of the concerned laboratory professionals in the existing CPD programmes.^19,32^ In addition to the reduced access to courses, there is also a lack of confidence in the quality of the training being conducted by many stakeholders.^19^ Continuous professional development programmes that are not well designed to address identified training needs and current programmes often result in low motivation for CPD.^30^

Studies have highlighted the qualitative benefits of CPD, emphasising its role in improving service delivery and patient outcomes, particularly in the fields of HIV and tuberculosis, which are of high priority in sub-Saharan Africa.^30,33^ However, there is limited published information on the implementation of CPD programmes in Africa. Additionally, because of limited mechanisms and infrastructure to maintain professional registrations, certification, licensure, and re-licensure, most of the laboratory workforce is not obliged or motivated to acquire or maintain relevant skills and competencies. The need for CPD in Africa is increasingly becoming critical, considering the threats that new microbes pose, as well as the rapidly evolving technologies being developed in response. Few national CPD providers that provide training for medical laboratory professionals exist across the African continent.^24,33^

As countries in Africa set up their own CPD programmes, harmonisation of these programmes across countries will be desirable and necessary to facilitate movement of laboratory professionals within the region as has been done in the European Union.^34^ This should also extend to in-country situations, where standardisation of CPD requirements and credit systems is done across all implementing in-country partners and regulatory bodies. Countries should make every effort to provide clear guidance to users and all providers of CPD and make sure that these guidelines for CPD development, implementation, monitoring and evaluation are clear.^1^

### Continuous professional development practice in Africa

Continuous professional development implementation for laboratory professionals in Africa presents a complex and varied picture. For instance, for the majority of African nations, the CPD activities predominantly fall under the umbrella of professional organisations. This includes a diverse array of entities such as medical associations, academies, professional bodies, government organisations, academic and training institutions, which are responsible for initiating, conducting, and promoting CPD programmes.^35^ The credit point system is integral to this structure, serving as a quantifiable measure of the time invested in CPD, thereby facilitating monitoring and record-keeping.^36^ However, a prevalent challenge is the potential reduction of CPD to a mere collection of attendance certificates, which may not necessarily reflect a genuine engagement with the content or an actual interest in the subject matter. Such a practice risks engendering a misleading sense of security regarding the true impact and outcomes of CPD efforts.^30^ It is imperative that CPD initiatives not only provide educational opportunities but also inspire real interest and relevance to the professionals’ practice to ensure meaningful professional growth.

### Research questions

This article aims at understanding how CPD training programmes for laboratory professionals are implemented and sustained in Africa. Specifically, it describes which African countries have mandatory CPD programmes and how they are implemented. We further explore the requirements that are essential for the effective deployment of CPD programmes in Africa. Lastly, we review what facilitates and hinders the implementation of CPD in Africa.

## Methods

This study employed a narrative review where a comprehensive search was conducted across PubMed, Embase, and web-based grey literature repositories, utilising a custom Python script to automate query execution across multiple databases.^37,38^ The review aimed to evaluate the effectiveness, challenges, and implementation of CPD training programmes in Africa. The search strategy included Boolean operators and Medical Subject Headings terms were tailored for each database to maximise relevant results, and it included: (‘continuous professional development’ or ‘CPD’ or ‘medical education’ or ‘professional training’). It also included ‘Africa’ or ‘sub-Saharan Africa’ or ‘low- and middle-income countries’ or ‘training effectiveness’ or ‘barriers’ or ‘evaluation’ or ‘capacity building’.

The inclusion criteria involved all peer-reviewed or grey literature published from 2009 to 2024. Studies evaluating CPD models, implementation, impact, or challenges within African healthcare systems were included. These were both quantitative and qualitative evaluations of CPD programmes. The following were excluded: commentary articles, non-evaluative literature, and articles not in English. The process involved **title/abstract screening**, followed by **full-text review**. Data extracted included study characteristics, CPD intervention types, training duration, effectiveness outcomes, and barriers identified.

### Experiences of continuous professional development programmes in Africa

#### Mandatory continuous professional development programmes and their implementation in Africa

Countries in Africa are at different levels of development for institutional support of promoting and encouraging human resource development through a CPD programme. While the actual training needs and gaps for CPD programmes are widely known – based on a few country surveys, the actual implementation of CPD has remained a challenge. For example, the full implementation of the policy for CPD in Nigeria that was established in 2012 is not yet well known. In Nigeria, it is noted that its implementation has been affected by lack of awareness by institutions and individual laboratory professionals themselves.^26^ Concerning training needs, an assessment of CPD needs in Nigeria showed that training on quality management systems was a priority. A study on laboratory professions in Botswana observed that the participants wanted CPD to focus on quality management systems, technical and practical cases, skills assessment and customer care.^10^ A study in Ghana also identified quality management systems as a priority focus for CPD programmes.^27^ These studies found that holding workshops, conducting practicals, and face-to-face presentations were the preferred mode of conducting CPD. Little to no reference in the studies was made about which institutions should be responsible for delivering the CPD programmes in Botswana.^10^

To address this training need on quality management systems, a continent-wide CPD training programme called Stepwise Laboratory Quality Improvement Process Towards Accreditation (SLIPTA) was launched by the World Health Organization’s Regional Office for Africa and other partners in 2011.^2^ The aim of the SLIPTA programme was to build quality systems for medical laboratories to ensure laboratories produce timely, reliable and accurate laboratory results.^39^ As of December 2023, over 500 African laboratories had been audited and awarded star ratings for the stage of their laboratory quality (using a 0- to 5-star award system) according to the SLIPTA programme.^40^ This programme has tremendously increased laboratory professionals’ knowledge and expertise in implementing quality management on the continent.

Another programme that was launched at the same time by World Health Organization’s Regional Office for Africa and partners to address quality issues was the Strengthening Laboratory Management Toward Accreditation (SLMTA) programme. The SLMTA is a laboratory training programme with practical activities designed to equip laboratory professionals with skills and competencies for establishing and building quality management systems in laboratories that are working towards general quality improvement and/or international accreditation. The difference between the two programmes is that SLIPTA is a tool for auditing laboratories while SLMTA is a training and mentorship toolkit.^39,41,.42,43,44,45,46^ Regional trainings on SLMTA were conducted and, in the process, created a grassroots movement on quality, as laboratory staff felt competent and empowered to improve their laboratory processes.

Despite the implementation of the SLIPTA and SLMTA programmes, challenges with achieving accreditation have persisted. A study in 2015 in Ethiopia and another study in Rwanda done in 2018 showed that inadequate skilled laboratory personnel in Ethiopia and lack of funding in Rwanda were found to be challenges in attaining accreditation and sustaining laboratory quality management systems.^47,48^ Laboratory accreditation and quality management systems are not usually covered during pre-service training.^49^ Laboratory professionals will therefore need ongoing CPD courses on laboratory management and quality management systems to overcome challenges with the accreditation journey. In one case in Rwanda, out of a total complement of 45 staff, only four staff members had CPD training on quality management systems.^48^ Availability and access to ongoing CPD training that supports laboratory professionals to improve quality systems towards accreditation will therefore be critical.

Experiences in Malawi, Tanzania and South Africa noted that non-governmental organisations and other partners continue to coordinate, provide, track and monitor CPD activities.^30^ However, this has led to some challenges with overall coordination and harmonisation of CPD activities being conducted by many different stakeholders. Most of the donor-funded CPD activities are usually fragmented and delivered to address disease-specific agendas that are designed around the goals and funding cycles of donors.^30^ In addition, these become a template across many countries and do not solve country-specific training needs.^30^ There is need for close involvement of countries, especially the recipients of CPD programmes, so that these trainings can feed into the overall capacity building targets and training goals.

Many African countries continue to struggle with the implementation of CPD training programmes. According to the 2015–2020 Health Sector Transformation plan in Ethiopia, the Federal Ministry of Health reported challenges in the implementation and actual realisation of the CPD programme in the country.^50^ This shortcoming on human resource capacity development affected all the health workers.^50^ Also, there have not been efforts to ensure that CPD activities are used for re-licensure of health professionals. In 2018, the Federal Ministry of Health took some policy decisions to ensure that the CPD programme gained traction.^50^ Unfortunately, the gap between the policy and CPD implementation remains. The same challenges observed in the other countries, such as lack of standardisation, and regulation of CPD programmes, continue to hinder progress in Ethiopia.^1,19^ In Ghana, a study was done to describe training needs of medical laboratory professionals; however, no institutions or programmes were identified to deliver these trainings.^27^

#### Essential requirements for effective deployment of continuous professional development programmes in Africa

According to several studies, limitations on utilisation of CPD range from time constraints, travel distance and perception of its importance.^10,27,28^ In addition, the format of CPD delivery has recently come into question. Lectures or seminars have been used for a long time as delivery methods for training and, thereafter, awarding of CPD points. However, CPD trainings are now being delivered in other ways, such as in-house lectures, short workshops, safety sessions, quality assurance programmes, and journal clubs.^10^ It has been shown that the delivery style of CPD is strongly linked to improvement of competence. For example, the educational formats that are engaging to participants (e.g. case discussion, role-play, hands-on practice sessions) can produce the desired learning outcomes as compared to didactic and formal instruction.^10^

The advent of online resources and learning opportunities have resulted in many professionals being able to conduct training in the comfort of their homes or in their offices. Over the last decade, the pace at which technology is changing in terms of patient management and diagnosis has led to professionals leaning towards online resources to be able to stay abreast. The online CPD programmes are considered to be cost effective as compared to being physically present at seminars or workshops.^51^ This has gradually changed the learning behaviour, especially as laboratory practitioners get more comfortable with digital platforms.^51^ Clinicians in sub-Saharan Africa have been shown to prefer online courses as compared to workplace or external locations – citing the convenience it brings for busy professionals.^29^ Slow and limited internet access, however, will remain a huge challenge to creating online CPD opportunities for most African countries. As such, a blended approach that utilises both virtual and in-person CPD trainings will remain important so as to fulfil all learning needs and preferences.

Laboratory professionals are most likely to benefit from CPD programmes that allow them to return to their workplace and practise what they have learnt.^30^ Most importantly, CPD should be accompanied by equipment and resources to enable professionals to put what they learnt into practice. This is not usually the case, as seen in a study among nurses in Malawi, Tanzania and South Africa, where apparent lack of test kits, microscopes and other equipment severely limited the ability to put CPD into practice.^30^ Additionally, this may result in discouraging laboratory professionals from participating in CPD programmes, as they will not see the value of such trainings. Where such trainings are happening, they are becoming theoretical exercises and just a fulfilment of trainings arranged without input from beneficiaries. Such scenarios demonstrate a lack of consideration for local priorities or individual needs. The picture is, however, different for CPD topics that are donor driven, as these will have facility-based resources that are programme specific. Funding for such CPD is readily available and inadvertently has excluded most professionals who may not be involved in these programmes. Currently, large public health programmes such as for tuberculosis and HIV are driving most of the CPD programmes on the continent.^30^ Countries will have to adapt and learn to leverage these resources to advance other CPD training needs.

#### Factors that hinder implementation of continuous professional development programmes

Many professional regulatory institutions in developing countries are now pushing for proper regulation and enforcement of CPD requirements for re-licensing.^10^ However, the efforts to have these requirements actually implemented often varies from country to country. For example, in Uganda, Botswana and South Africa, health professional boards will not register professionals without a certain number of CPD credits.^10^ In other African countries, these requirements for re-licensing are not yet mandatory and therefore many laboratory professionals do not need to attend CPD trainings nor are they required to document any CPD trainings they do attend.^10^ In 2021, the African Society for Laboratory Medicine (ASLM), through a CPD project called ‘Sustaining Antimicrobial Resistance (AMR) Surveillance Qualification Framework’ conducted a landscape analysis on available CPD providers in 14 African countries.^52^ This allowed insights into the current status of workforce regulation, competency requirements and the associated CPD across the 14 African countries (Table 1). It is important to note that although 12 of the countries have documented guidelines on CPD requirements for laboratory professionals, the implementation of these requirements is still a work in progress, as most countries are still developing platforms to deliver accredited CPD programmes. Without funding and technical support, the countries will not be able to implement these CPD programmes.

**TABLE 1:** Regulatory councils and professional associations involved in continuous professional development in 14 African countries, October 2021.

Country	Name of regulatory council for laboratory professionals	Name of professional association	Re-licensing
Cameroon	National Order of Physicians of Cameroon (ONMC) Cameroonian Society of Clinical Biology (SCBC)	Cameroon Association for Medical Laboratory Scientists	Accrue 24 CPD points annually
Eswatini	Eswatini Medical and Dental Council	National Laboratory Association of Eswatini	Accrue between 15 and 40 CPD points, depending on the professional level, and pay annual licence fee Med Lab Technologist – 25 CPD points Med Lab Tech/Scientist – 30 CPD points
Ethiopia	Continuing Professional Development Committee of the Ministry of Health	Ethiopia Medical Laboratory Association	Accrue 30 CPD points per year and 150 credit units per five years to get a re-licensure
Gabon	None/NA	None/NA	None
Ghana	Allied Health Professional Council	Ghana Association of Medical Laboratory Scientists	Accrue 10–20 CPD points, depending on qualification and cadre
Kenya	Kenya Medical Laboratory and Technologist Board	Association of Kenya Medical Laboratory Practitioners	Accrue 50 CPD points per calendar year
Malawi	Medical Laboratory Council of Malawi	Malawi Association of Medical Laboratory Scientists	Accrue 25–40 CPD points, depending on qualification and cadre
Nigeria	Medical Laboratory Science Council of Nigeria	Association of Medical Laboratory Scientists of Nigeria	Accrue mandatory annual points; 10 CPD points are required for Medical Laboratory Scientists prior to licence renewal
Senegal	None/NA	None/NA	None
Sierra Leone	College of Medicine and Allied Health Sciences	Sierra Leone Medical Laboratory Association	None
Tanzania	Health Laboratory Practitioners Council	Medical Laboratory Scientists Association of Kenya	Accrue minimum of 20 CPD points for yearly renewal of licence
Uganda	Allied Health Professionals Council of Uganda	Uganda Medical Laboratory Technology Association	Accrue 50 CPD points
Zambia	Health Professions Council of Zambia	Zambia National Laboratory Association	Accrue 100 CPD points
Zimbabwe	Medical Laboratory and Clinical Scientist Council	Zimbabwe Association of Medical Laboratory and Clinical Scientists	Accrue 10–20 CPD points, depending on qualification and cadre

*Source*: African Society of Laboratory Medicine. Sustaining AMR surveillance qualification framework in 14 Fleming Fund priority African countries. 2021. Unpublished African Society of Laboratory Medicine report.

CPD, Continuous professional development; NA, Not applicable.

This CPD project on the qualification framework aimed to equip the AMR surveillance workforce (microbiologists and epidemiologists) with the knowledge and skills to be able to implement key functions of the National AMR surveillance system and activities under the ‘One Health’ approach across 14 priority African countries and three Asian countries.^52^ To explore options for continuity and sustainability of the CPD Qualifying the Workforce for AMR Surveillance in Africa and Asia (QWArS) qualification framework post-project implementation, ASLM engaged the country CPD stakeholders, including those involved in training of laboratory and epidemiology professionals in the priority countries.^52^ All the countries (14/14) indicated that they will recognise the CPD QWArS professional qualification. Of these, 6/14 (43%) were likely to also recognise the University of Witwatersrand CPD mechanism associated with the course, while 8/14 (57%) have more advanced implementation of national CPD programmes and are more likely to review, adopt and adapt the QWArS curriculum and assign their own CPD points.^52^ By adopting the CPD QWArS professional qualification and establishing roles for the QWArS qualified Trainees and Master Trainers, it is envisaged that the participating countries will access CPD of their AMR Surveillance workforce. With a pool of qualified personnel, the countries will be better equipped to implement their National Action Plans for AMR Surveillance to address the increasing threat from AMR at national, regional and global levels.^52^

Professional qualification and experience play a role in CPD attendance. In many developing countries, experiences show that differences in basic qualifications will also present unequal availability and opportunities to CPD programmes for laboratory professionals.^31^ It is usually the most qualified and those with many years of experience that get these training opportunities.^30^ The newly recruited professionals, even though in most need of CPD training, are usually left out of these opportunities; supervisors attend most CPD training as they happen to get the invitations and have the deciding power on who attends.^30^ This vicious cycle continues as in most institutions there are no training or capacity-building plans that have been developed and followed through.^30^ In other instances, the same individuals are repeatedly given the opportunity to attend CPD programmes, thereby demotivating others in the process.^30^ Other barriers for laboratory professionals interested in participating in CPD include workplace locations that are remote and rural. Unfortunately, these remote locations have the majority of the laboratory personnel who could benefit from the CPD programmes.

Health workers who are normally posted in rural and often neglected places face challenges related to travel distance, as well as a lack of communication and updates on upcoming CPD training opportunities.^19^ In some settings, laboratory professionals cannot take time to attend CPD training largely because there are only a few staff available to remain working in their absence.^24^ This situation therefore leads to inconsistent participation in CPD programmes.^24,33^ Organising CPD training workshops where participants are required to come to a central site or a particular country has been the norm for a while in many settings. This approach of travelling individuals is, however, proving to be expensive and not sustainable. As such there is limited training that can be delivered and may not be sufficient in addressing the training gaps. The extensive travelling logistics, especially for participants attending from different countries, adds to the challenges of in-person CPD training programmes.^24^ It is therefore imperative that administrators of CPD programmes consider alternative and effective approaches to delivering CPD training at low costs.

The current training packages designed and offered to increase the knowledge and skills of the recipients continue to face challenges with uptake. Instead of learning and skills development being owned and driven by the professionals themselves, learning is currently happening through a prescribed type of approach to training (top-down approach) and this has limited effects.^18^ Decentralising CPD programmes to increase their reach in Africa is another approach that can be promoted. Workplace CPD training opportunities could be adopted for rural and remote settings to improve opportunities – reducing barriers of distance and time.^53^ However, experiences from Ethiopia indicate that there is hesitancy to support the decentralised provision of CPD training programmes. Most laboratory professionals are uncomfortable with decentralising the CPD training programmes as they believe this will lead to poor quality and not achieve the intended goals of the programme. They also fear the possibility of enhancing illegal certification with a decentralised approach.^19^ Hence, many CPD programmes remain centralised and coordinated by established institutions.

To scale up training on the continent and create a critical pool of well-trained laboratory professionals with the aim of improving laboratory quality, several critical factors need to be addressed in any CPD approach. Addressing these factors will require recognition that individuals prefer an environment of continuous education that has a flexible format, relevant content and is delivered at a convenient time, as participants have many other commitments. They prefer to drive their own learning, especially that which is applicable to their life and work environment. Lastly, CPD programmes will work well when the credit system is familiar and understood by the professionals. This is because participants can calculate the CPD points they can earn and, as a result, are able to chart their learning journey to achieving the requirements on credit units.^54^ As CPD training programmes become more and more available, laboratory professionals will need to avoid attending trainings that are just a fulfilment of a requirement. First and foremost, a clear evaluation and understanding of the actual training needs will be important. Secondly, defining what trainings could be done internally and what can be outsourced or conducted externally to the workplace will be critical in establishing sustainable CPD programmes that are accessible in all locations.^55^

There are few studies that have defined and documented specific CPD training needs in Africa. Study results in Botswana and Ethiopia indicate that the preferred training areas for attention include technical, laboratory management, and leadership skills.^10,28^ This is not surprising, given the ever-changing technology and testing needs that were experienced because of emerging and reemerging diseases such as coronavirus disease 2019. The testing needs for the 21st century have become more complex with the increasing disease burden on the continent. Another topic identified is laboratory quality management systems.^28^ This interest in quality management systems has been created because of the scale-up of accreditation programmes on the African continent. The recognition and pride that comes with working in an accredited laboratory has spurred the demand for such trainings. National laboratory quality programmes have been initiated across the continent and many of these have been heavily funded by donors; hence, the increased interest and trainings. Interestingly, in Ghana, it was observed that laboratory professionals from the private sector preferred topics on quality management systems while those from public health facilities prioritised topics on technical areas.^27^ It is important to note that many CPD training opportunities have focused on the public sector professionals while ignoring private sector health workers.^28^ This can be attributed to the predominant nature of donor funding that is geared towards the public sector. Scaling CPD training programmes in Africa will require an all-inclusive approach, as the private sector also serves a huge population in Africa.

Another important aspect has been whether CPD can improve the professional’s working environment. Studies have observed that job satisfaction and retention are increased when well-designed CPD programmes are made available to professionals.^56^ Without increased opportunities for CPD, laboratory professionals may become demotivated and experience restricted career growth.^56^ Laboratory professionals who have been upskilled, and therefore have certain opportunities because of additional competencies, are more likely to be happy and remain in their jobs. Currently on the continent, health workers struggle to find the right CPD programmes for their career growth. This is compounded by a lack of sufficient data, experience and guidance on how to design and implement relevant and strong CPD programmes.^57^

As already noted, one of the persistent barriers for many CPD programmes on the continent is funding. The scale, quality, effectiveness and reach of these CPD programmes will hinge on the availability of resources. On one hand, you have individual laboratory professionals without funding and resources to attend CPD opportunities. On the other hand, institutions are struggling to support, sponsor and meet some of these CPD training goals.^30^ Mulvey adds a third component that is critical in the implementation and funding of the CPD programme – the employer.^17^ In what the author calls the CPD ‘triad’, namely the individual, the institution and the employer, the author argues that the employer is important, as the CPD programme costs money and time off work.^17^ The employer will have to understand that an up-to-date, well-trained and efficient employee will increase productivity and confidence in the product or service being provided. It is therefore worthwhile for employers to invest in CPD.^17^ Unfortunately, the prevailing status is that most of the CPD programmes in Africa have been funded by donors, local and international non-governmental organisations, with less support coming from governments.^30^ In Ethiopia, this has been attributed to lack of commitment from upper-level management in supporting CPD trainings, citing cost issues.^19^ In a survey in Nigeria, almost 90% of the interviewed participants indicated lack of funding to attend CPD training programmes and, as result, there was low attendance for CPD training.^26^ Therefore, lack of sustainable financial resources is a huge barrier to CPD planning, implementation, evaluation and continuity.^30^

A new CPD programme launched under the ASLM Academy is envisioned to play a pivotal role in restructuring CPD in Africa by addressing the implementation challenges.^58^ The ASLM Academy is expected to implement a continent-wide standardised scoring system that recognises diverse learning activities and accommodates the varied needs of professionals. The ASLM Academy will create and distribute comprehensive learning materials that are accessible even in low-resource settings, providing a mix of online and face-to-face training options to cater to different learning preferences and connectivity issues.

## Conclusion

In summary, implementation of CPD programmes in Africa remains a challenge. Linking CPD training to licensure and implementing mandatory CPD programmes will be critical in developing a well-skilled and up-to-date laboratory workforce.
